# Combined Use of Terrestrial Laser Scanning and IR Thermography Applied to a Historical Building

**DOI:** 10.3390/s150100194

**Published:** 2014-12-24

**Authors:** Antonio Costanzo, Mario Minasi, Giuseppe Casula, Massimo Musacchio, Maria Fabrizia Buongiorno

**Affiliations:** 1 Istituto Nazionale di Geofisica e Vulcanologia, Centro Nazionale Terremoti, Via P. Bucci Cubo 30c, I-87036 Rende (CS), Italy; E-Mail: mario.minasi@ingv.it; 2 Istituto Nazionale di Geofisica e Vulcanologia, Sezione di Bologna, via Donato Creti 12, I-40128 Bologna, Italy; E-Mail: giuseppe.casula@ingv.it; 3 Istituto Nazionale di Geofisica e Vulcanologia, Centro Nazionale Terremoti, Via di Vigna Murata 605, I-00143 Roma, Italy; E-Mails: massimo.musacchio@ingv.it (M.M.); fabrizia.buongiorno@ingv.it (M.F.B.)

**Keywords:** terrestrial laser scanner, IR thermography, historical buildings conservation, preventive diagnosis

## Abstract

The conservation of architectural heritage usually requires a multidisciplinary approach involving a variety of specialist expertise and techniques. Nevertheless, destructive techniques should be avoided, wherever possible, in order to preserve the integrity of the historical buildings, therefore the development of non-destructive and non-contact techniques is extremely important. In this framework, a methodology for combining the terrestrial laser scanning and the infrared thermal images is proposed, in order to obtain a reconnaissance of the conservation state of a historical building. The proposed case study is represented by St. Augustine Monumental Compound, located in the historical centre of the town of Cosenza (Calabria, South Italy). Adopting the proposed methodology, the paper illustrates the main results obtained for the building test overlaying and comparing the collected data with both techniques, in order to outline the capabilities both to detect the anomalies and to improve the knowledge on health state of the masonry building. The 3D model, also, allows to provide a reference model, laying the groundwork for implementation of a monitoring multisensor system based on the use of non-destructive techniques.

## 1. Introduction

Today, local communities and civil societies give an increasing importance to their historical and cultural heritage, showing a great interest in their preservation and conservation. In this framework, were promoted the drafting of specific legislations; for example, the Italian Code requires a precise geometrical survey to know the building in details and to detect the anomalies and morphology of the surfaces, in order to set up a highly realistic structural model. Moreover, the same Code attributes a great importance to planning of an appropriate monitoring program, for example following consolidation works, controlling in the time cracks, displacements (absolute and relative) and rotation of structural elements. Therefore, the use of non-destructive and non-contact techniques is strongly recommended, in order to allow the acknowledgement of the conservation state of the built heritage and, at the same time, to preserve its integrity.

As the evaluation of the conservation state of historical buildings using destructive techniques should be avoided to preserve the integrity of the cultural heritage, the development of non-destructive and non-contact techniques becomes of crucial importance. Currently, although several non-destructive techniques are available, there is an increasing demand of instruments with greater reliability, sensibility, user friendliness and high operational speed.

In the process of analysis and diagnosis of the conservation state of architectural monuments, the first step is represented by the acquisition of geometrical data. It allows to define a reference basis according to which the other further information derived from different disciplinary fields must be referred, in order to achieve historical, architectural and experimental integrated knowledge of the monumental asset. For this purpose, the terrestrial laser scanning technology is used to acquire dense point clouds defined by spatial three-dimensional coordinates in order to reconstruct a high-resolution representation of the target, permitting the real-time access to an automatic and non-contact geometric measurement [1]. Also, the reconstruction of a high accurate 3D model allows to perform the checks of the residuals on the buildings and to define a reference system for the monitoring of structural modification and damaging [2].

Instead, the use of infrared thermal (IRT) imaging is a powerful tool for inspecting and performing non-destructive testing of building elements. The analysis of thermographic data can allow to identify anomalies that could otherwise go undetected before they evolve into damages of the structure [3]. Nevertheless, the diagnostics, that is to identify the causes of damage and decay on the basis of the acquired data, is often a very complex process; in fact, the available data usually refer to the effects, while, it is more difficult to identify the causes that have produced the damage. Due to the simplicity of operation and the reduced cost of several thermal cameras with low geometric resolutions, to date IRT is popular among professionals working in building maintenance. However, most the IR sensors are capable of capturing small rectangular images, suitable for localized investigation in restricted areas, as single image is sufficient to obtain the information on suspect pathologies; instead, in the case of large constructions the independent analysis of single images is not enough. In the last case, images have to be mapped on the surfaces to be analyzed. This operation, usually called texturing, requires a 3D model of the object [4].

In this framework, methods for the combination (*i.e.*, comparison and overlay) and fusion (*i.e.*, integration of all data in one data set) of data obtained by both thermography and 3D laser scanner have recently been developed for the monitoring of damaging, restricted to single elements [5]. More often in the professional practice the two sensors were used separately, although some research have been made to study instruments and methods for overlaying data on whole parts of buildings [4,6]. Therefore, the two sensors are used in a separate way decoupling the use of TLS for 3D reconstruction and IR thermography to detect the thermal anomalies.

Within a research project aimed to realize a monitoring system of monumental buildings in seismic areas, a working group, including the authors, has the task to reconstruct the 3D models and to perform the thermographic surveys of some historical buildings located in different provinces of the Calabria Region. In the presented work the TLS was also used as diagnostic tool to study the pathologies and to evaluate the vulnerability of a historical building. Combined analyses of data obtained by both sensors allowed to assign the metric features to the thermographic images and, consequently, to mapping the possible anomalies, making them measurable. Furthermore, overlaying the different characteristics a better understanding of the kind anomaly was obtained, in particular when the thermal defect was associated with geometric irregularity. At this purpose, a methodology was designed not only to reconstruct the 3D model, but also to detect the anomalies and to analyze the vulnerability of the structural elements. The obtained results could be used to improve the knowledge of the conservation state of the ancient buildings and to check the structural modeling. In the paper, is presented the adopted methodology and its application, with particular reference to a specific case study.

## 2. Instrumentation

### 2.1. Background on Terrestrial Laser Scanner (TLS)

The introduction of advanced terrestrial laser scanner devices in the survey field has increased the possibility to obtain more accurate and complete 3D models. This happens, especially, in the architectural and archeological survey field in which the shape of an object is usually remarkably complex. Combining the distance with the two internal angle measurements of the rotating mirrors of the scanner, a spherical coordinate system centered on the scanner can be defined and any point on the surface of the object can be recorded using this spherical coordinate system. The set of coordinates representative of a scanned object is a so-called point cloud. Generally, the scanner allows to detect for each recorded point two kinds of information: the position, as set of coordinates, and the reflectance, as ratio between the emitted and reflected phases (or energy) of laser wave.

In the present work the TLS survey was executed using a Z+F Imager^®^ 5010c laser scanner (Zoller & Fröhlich, Wangen im Allgäu, Germany) based on the method of phase comparison e.g., [7] with wavelength of 1.5 μm, therefore a laser of class 1 following the UNI EN 60825-1 code. The sensor can be used on the distance range from target between 0.3 m and 187 m, acquiring over a million points for second, consequently with a measurement accuracy beneath 1 mm for distance of few meters. The Z+F Imager^®^ 5010c is also equipped with the high dynamic range (HDR) camera capable of generating panoramic pictures characterized by resolution of 80 Mpixel.

Although the campaign activities can be quickly performed by a staff with adequate experience, however, particular attention must be paid during the analysis, the processing and the modeling of the data. Indeed, the point clouds can be often characterized by the presence of high noise, which must be removed by *ad hoc* techniques before starting with the manipulation of the data. Furthermore, usually architectural and archaeological objects have a very complex shape and one scan is not enough to obtain the complete description of the scene. In these cases, in order to eliminate the shaded areas, more scans must be taken from different points of view of the same object; accordingly, an alignment of the single scans is necessary to obtain the 3D reproduction, thereby introducing an intrinsic error in the model due to this processing phase. When it is necessary to match a large series of scans in a unique reference system, initially an automatic or manual scan to scan pre-alignment is performed setting at least three reference points detected in both reference and mobile (free to shift and rotate) clouds, afterwards an interactive closest point algorithm is implemented, for example based on the least squares method, to avoid significant distortions of the 3D model [8,9]. In this work, the filtering for noise reduction, the alignment of the point clouds and the analysis of the data have been made using the JRC 3D Reconstructor software [10].

### 2.2. Background on InfraRed Thermography (IRT)

Infrared thermography is a powerful non-destructive and non-contact diagnostic technique based on the measurement of the heat energy and its conversion into an electrical signal, which is represented by thermal digital image. The operating principle of a thermal sensor is explained by the well-known Wienɿs displacement law:

$$\lambda_{\text{m}}=2897.8/\text{T}$$

where λ_m_, wavelength of maximum spectral radiant emissivity expressed in μm, and T, absolute temperature in K, result inversely proportional. The devices are calibrated to measure the emissive power in an area at various temperature ranges, therefore they work in a wavelength range included in the IR spectrum. The temperature of the target is estimated by the emissivity (*i.e.*, the ratio between emitted radiation by a surface and that by a blackbody at the same temperature), therefore the behavior of the detected objects is approximated to the model of the gray body, characterized by the emissivity less than unity and constant respect to wavelength and temperature e.g., [11,12]. In order to focus the emitted IR radiation onto a detector is used an appropriate lens and the electrical response signal is converted into digital picture in which the different colours correspond to various temperature levels of the target surface. The thermal images can be analyzed by specific software to quantify the difference between temperatures [13]. The accuracy of the measurement depends on different parameters: ambient temperature, rain, wind speed, distance from the target. However, any factor, which produces changes in temperature, actually can help in identifying the anomalies and the features e.g., [11].

The advantages of using a thermal sensor to measure the surface temperature are: remote sensing, two-dimensional data acquisition, rapid response, non-contact, high resolution, large temperature range, versatility and portability [14].

With reference to the study presented in the paper, the thermographic surveys were conducted by the authors using the Avio (NEC) Thermal Imager R300SR-S (Nippon Avionics, Tokyo/Yokohama, Japan). The device is characterized by a maximum wavelengths ranging from 7.4 μm to 12.4 μm, corresponding to measuring range of emitted temperature between −40 °C and 120 °C, and a resolution of 0.03 °C at 30 °C of environmental temperature [15]. Moreover, the instrument in the super resolution (SR) mode enables to capture a image with pixel definition of 640(H) × 480(V) and an instantaneous field of view of about 0.8 mrad.

The recorded images by thermal camera have been processed and analyzed using the NRG software [16], permitting to define the temperature and to show the distribution on the building surfaces, according to the thermal properties of structures and their defects.

Although the influence of emissivity coefficient is relevant to measure the correct temperature of a surface, if the study aims to detect the anomalies on a building, through an analysis of the superficial temperature differences, the adopted value for this parameter is not very influential [17,18]. Furthermore, typical value for the construction materials varies between 0.88 and 0.94 [19,20]; in this study the emissivity coefficient was set to a mean value of 0.91 for all the illustrated thermal images.

## 3. Case Study and Methodology

**Figure 1.**
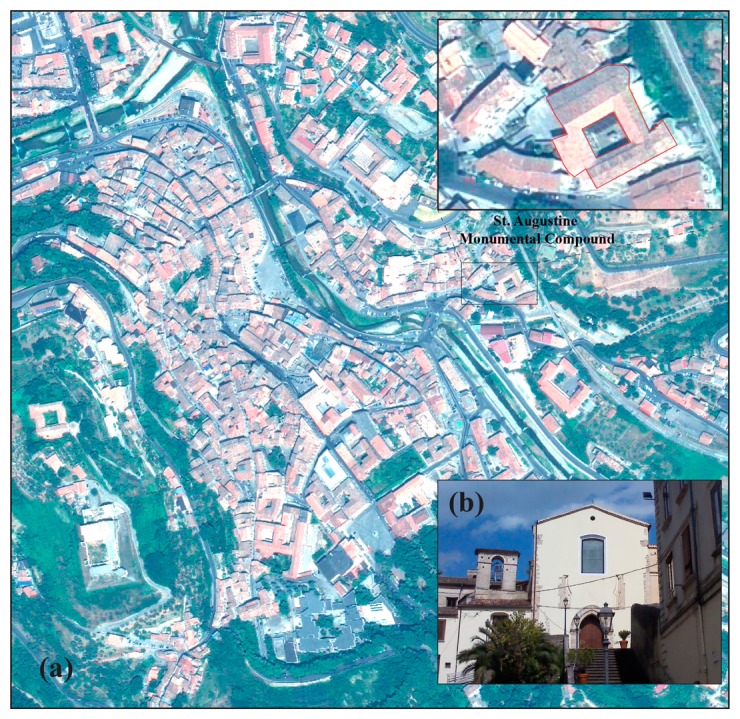
Historical centre of Cosenza indicating the location of (**a**) the St. Augustine Monumental Compound and (**b**) a picture of the church façade.

Located in the historical centre of Cosenza on the right side of the Crati River, the St. Augustine Monumental Compound is constituted by the homonymous church and the adjacent monastery (Figure 1), transformed into the Museum of Bretti and Enotri since 2009.

**Figure 2.**
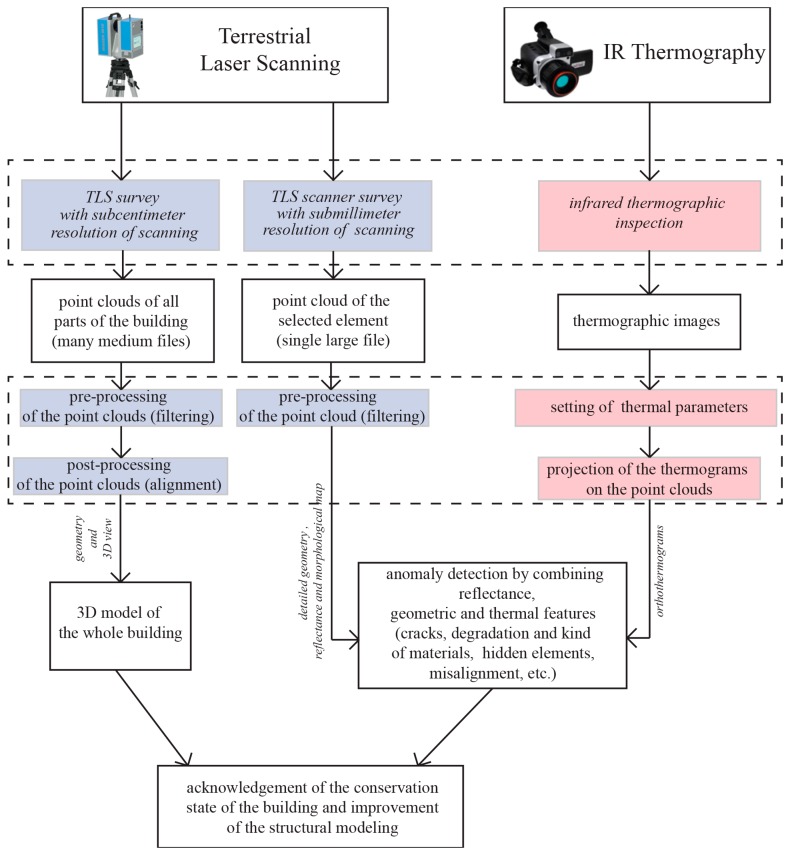
Scheme of the methodology used to integrate the results of TLS and IR thermography, in order to improve the knowledge of the conservation state of historical buildings.

The monastery was built in 1507 by friars of the Augustinian mendicant order, in the ancient village of Pignatari. The church had suffered strong damage, first as a result of strong earthquakes (likely the Calabria earthquakes of 1638, but also from subsequent major seismic events in 1854, 1870 and 1905) and then by a fire in the XVII century (1640). Only after about a century was the religious building restored varying the original features. Since 1810 the monastery suffered different transformations, being variously used as a military station during the Napoleonic domination, as a prison during the Bourbon one and as a shelter for displaced persons in the Fascist period. The Bandiera brothers, famous Italian patriots, were held captive in the St. Augustin compound up to their execution and then they were buried in the church for some years. This brief history demonstrates the great importance of the building in the cultural and architectonical heritage of the town and, more broadly, for the Calabria Region, which has led it to become an important civic museum. Today the façade of the church still preserves some feutures from its foundation that can be observed in Figure 2, in particular the lancet arch above the entrance, two decorative columns in the central part and the bricks left exposed corresponding to the lateral edges. The historical and architectural analysis has highlighted the likely presence of a rosette, instead of the window, surrounded by the abovementioned columns and arch [21].

The conceived methodology, involving the coupled use of TLS and IRT (Figure 2), was applied to the ancient monumental compound, in order to highlight the anomalies and to detect its vulnerability. In the survey phase, the TLS was used such to define an accurate geometry of the building, setting “high resolution” of instrument (3.2 mm to the distance of 10 m between receiver and target) and obtaining scans characterized by resolution at least one centimeter, as to carry out the detailed survey for evaluating the anomalies on the surfaces, with “super high” or “extremely high” resolution of the sensor (1.6 mm or 0.6 mm to the distance of 10 m between receiver and target), obtaining millimeter or sub-millimeter resolution of the scan depending on the distance.

The data processing phase was divided in two different stages: the pre-processing, consisting of data filtering, and the post-processing, aimed to align more scans. In fact, for the reconstruction of a whole historical building, characterized by a complex shape and lots of elements, the alignment of more point clouds is often required, introducing an intrinsic error due to the distortion of data. Therefore, with reference to the purpose of acquisition, also in the processing phase of data two assessment procedures were identified. In fact, in order to reconstruct the 3D model, both stages of data processing were carried out; conversely, the detailed analyses were performed on the basis of single scans, to avoid invalidation of the extremely high resolution used during the surveys. These analyses were aimed to highlight cracks, degradation and kind of materials, rotation and misalignment of the selected elements, therefore a more detail. Furthermore, the acquired point clouds allowed evaluation of the residuals with respect to a fitting plane of the scanned target adopting an area-based method; in particular, the check was carried out by comparison between acquired scan and mathematical surfaces, represented by planes fitting the measured points [22]; moreover, in this way the surface, interpolating the extended group of selected points, could act as filter on measured noise [23]. With regards to the validity of the deviations respect to a fitting plane used as indicator of non-homogeneity or anomaly detectable on the construction materials, Casula *et al.* [24] and Fais *et al.* [25] demonstrate the reliability of this analysis comparing the TLS data and acoustic techniques. In some research works available in literature, this application has been tested to monitor the displacements over time, comparing more geo-referenced scans respect to the same mathematical surface e.g., [23].

With reference to the case study, ensuring the scanning resolution established in the procedure, the compound was scanned by TLS, at first, setting “high resolution”, resulting need fifty scans to cover the entire building; afterwards, imposing the “super high” or “extremely high” resolution, were scanned selected elements, having care to reduce the acquisition distance compatibly with surrounding logistic and environmental conditions.

In the processing phase of TLS data, all point clouds were pre-processed through the application of both median and mixed point filters in order to reduce the environmental noise and the parts of the scans not related to the studied building were eliminated. Instead, only for the 3D reconstruction of the entire compound, the point clouds, colored by the HDR photographs, were pre-aligned two by two though semi-automatic procedure, identifying different target points in both scans, and, at a later stage, these were submitted to the alignment using an interactive closest point algorithm, based on the least squares method, implemented in the latest version of the JRC 3D Reconstructor software. At the end of the alignment phase a mean error of about 2 mm was introduced in the model. To verify this model other open source or commercial software were also used for the alignment of all point clouds.

Following the procedure, where the distance map detects geometrical irregularities, thermographic surveys were performed to associate any thermal defect. After setting the parameters relating to thermal image (emissivity, temperature of background, *etc.*), in order to overlay the geometric and thermal data, ensuring the correspondence between based-TLS model and the thermal images, the camera calibrations were executed. For each thermogram, external parameters (position and orientation relatively to the coordinate system) and the internal parameters of the camera (image centre, focal length and distortion coefficients) were determined with respect to the reference scan. The calibration technique, adopted in this work, was the one proposed by Tsai [26], its implementation needs to verify at least 11 correspondences between 3D point coordinates and 2D pixels in the image for computing external and internal parameters of the camera and for generating the orthothermogram.

## 4. Experimental Data and Discussion

In about one year, different measurement surveys at the test building were conducted, with the main purposes to reconstruct the 3D model, using TLS, and to verify properties and conditions of structural and architectonical elements, combining detailed data by TLS and thermal images.

### 4.1. Geometrical Surveys

**Figure 3.**
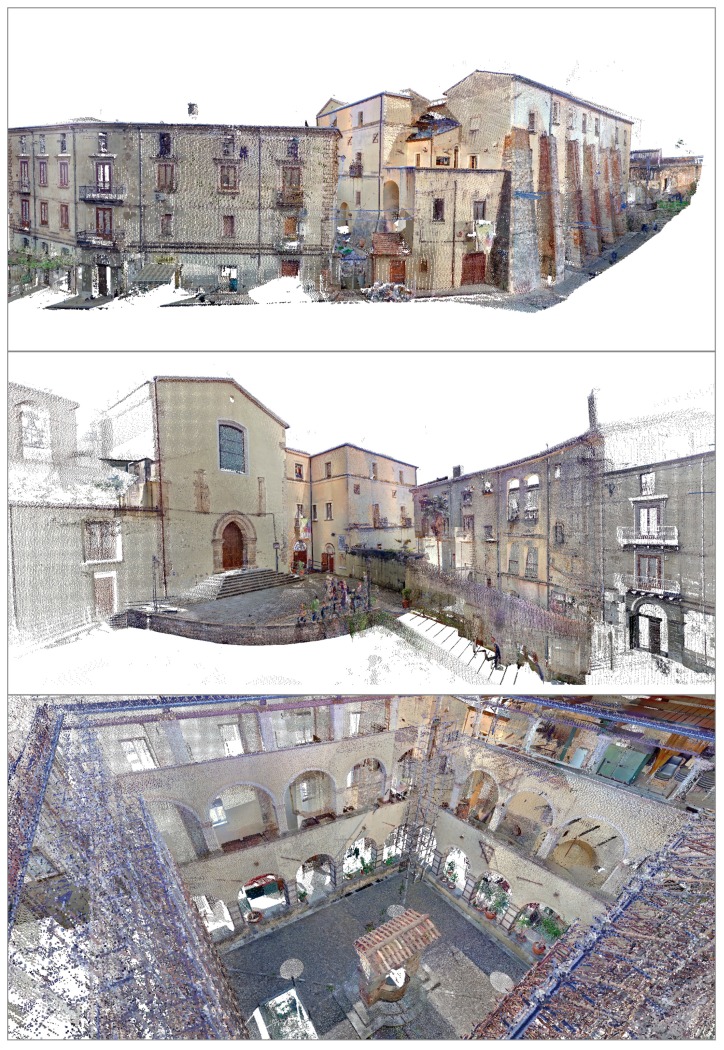
Views of the tridimensional model of the St. Augustine monumental compound obtained by alignment of the scans.

The geometrical surveys were executed with “high resolution” of the laser scanner sensor, ensuring a scan resolution of at least one centimeter. In the examined case, fifty scans were performed to cover as many parts as possible of building, with particular attention to overlay some targets of each acquisition to the previous. Using the JRC 3D Reconstructor software all scans were filtered and the points not related with the building were eliminated from the clouds; afterwards, during the post-processing the scans were aligned, limiting the mean registration error to a value of about 2 mm. At the end of this phase more than 320 million of points were obtained to represent the entire compound. Subsequently, the data were subsampled to allow faster management and also to obtain a 3D skeleton of the reference building in order to achieve a virtual modeling and to identify the measurement points of the different non-destructive techniques, as provided in the research project. Figure 3 shows some views of the observed area through the arrangement of all point clouds. The TLS surveys allowed us to realize a complete model of the historical compound, necessary to assess the correct spatial position and the geometric dimension of all structural elements, aimed also to improve the finite elements model for the structural analysis.

Furthermore, some architectural and structural elements of the building were reconstructed by meshing on the basis of the acquired data. An example is the ancient well located at the centre of the inner cloister.

**Figure 4.**
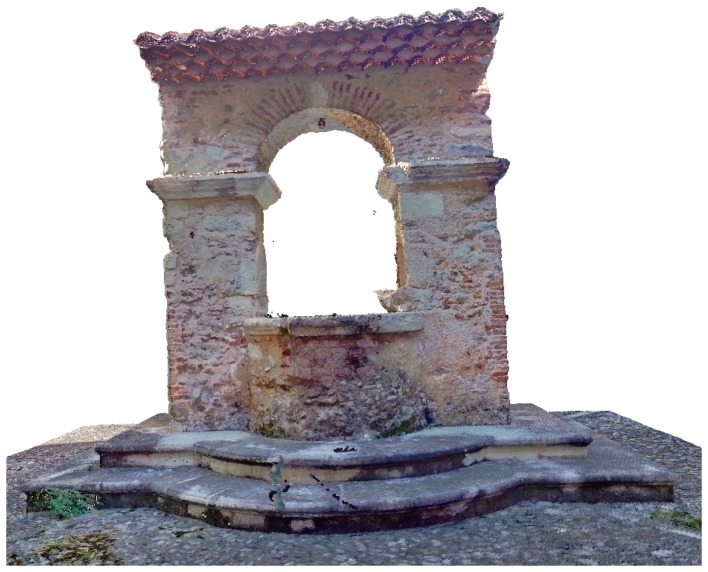
Tridimensional mesh of the well inside the cloister of the compound.

Figure 4 reproduces this functional element from the monastic life built of chaotic masonry, composed by coarse aggregates and brick-faced; all materials were reconstructed in 3D modeling with good approximation. The well, 3.15 m high and 2.81 m wide, is adorned by a rounded arch supported through two columns with square base of side about 0.70 m and decorated with capitals, making it an appreciable architectural element.

### 4.2. Detailed and Thermographic Surveys

The detailed analysis was carried out by scans obtained with the “super high” or “extremely high” configuration of the TLS sensor, in order to obtain millimeter or sub-millimeter resolution of the point clouds depending on the acquiring distance. To better highlight the real configuration of the surfaces, providing also the point-to-plane variations at a large scale and to show any deviation, local best-fitting planes were created using point cloud subsets. Moreover, where the maps of the residuals showed areas of anomalies, was performed also the thermographic survey.

Due to its historical relevance as the oldest part of the compound, the most important experimental efforts were spent to analyze the façade of the St. Augustine church. In a previous study available in literature (*i.e.*, [27]) on the basis of mineralogical and petrographic analysis, it is possible to observe that the church is constituted exclusively by local stone materials, known as Pietra di Mendicino, which was abundantly extracted from several quarries in the surrounding area. Moreover, the tests conducted on different samples of the mortar have allowed verification of the various construction phases of the building, as reported by the historical survey [21].

**Figure 5.**
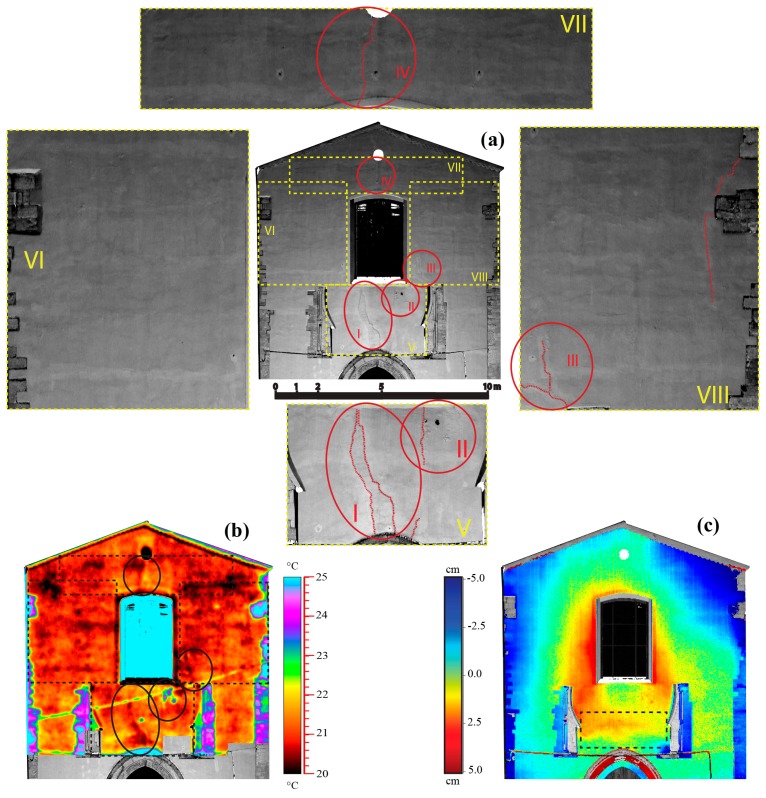
Comparison between terrestrial laser scanning and thermographic survey on the façade of the St. Augustine church: (**a**) 3D mesh obtained by the point cloud in terms of reflectance and details; (**b**) thermal image projected over the mesh and (**c**) map of residuals with respect to a fitting plane.

In order to verify any anomalies, the external façade of the St. Augustine Church was investigated comparing the information detectable directly by the reflectance point cloud (Figure 5a), carried out by thermographic surveys (Figure 5b) and analyzing the data acquired by terrestrial laser scanning (Figure 5c).

Through the examination of the results is possible to recognize:

- in the central part, a large crack linking the lancet arch to the base of the window; this defect, barely visible to the naked eye, contrariwise becomes easily detectable from both the 3D scan and thermographic image (Figure 5a,b—zone I);
- from the reflectance map, minor cracks: one that links the right column with the respective corner at the base of the window (Figure 5a—zone III), another that connects the top of the window with the upper circular opening (Figure 5a—zone IV) and still others that are indicated in frame VIII; as noted in the previous case, in the thermogram these cracks are characterized by lower temperature than the surrounding areas;
- below the window, on the right side, the swelling of the plaster, which appears partially detached (Figure 5a—zone II), this exposed area is characterized by a temperature comparable with that shown by the other elements without plastering (cf. Figure 5b), like the columns above the portal and the stones on the lateral edges of the façade (cf. Figure 5a,b);
- in correspondence to the zones identified from VI to VIII frames in the Figure 5a, horizontal imperfections directly seen on the reflectance map; the analysis of the thermogram makes clear that some part of these zones are characterized by the lowest temperature on the façade (black zones in Figure 5b). It is worth emphasizing that these imperfections are detectable from the base of the windows to the roof of the church, which should be the part of the façade subjected to the reconstruction work over time.

**Figure 6.**
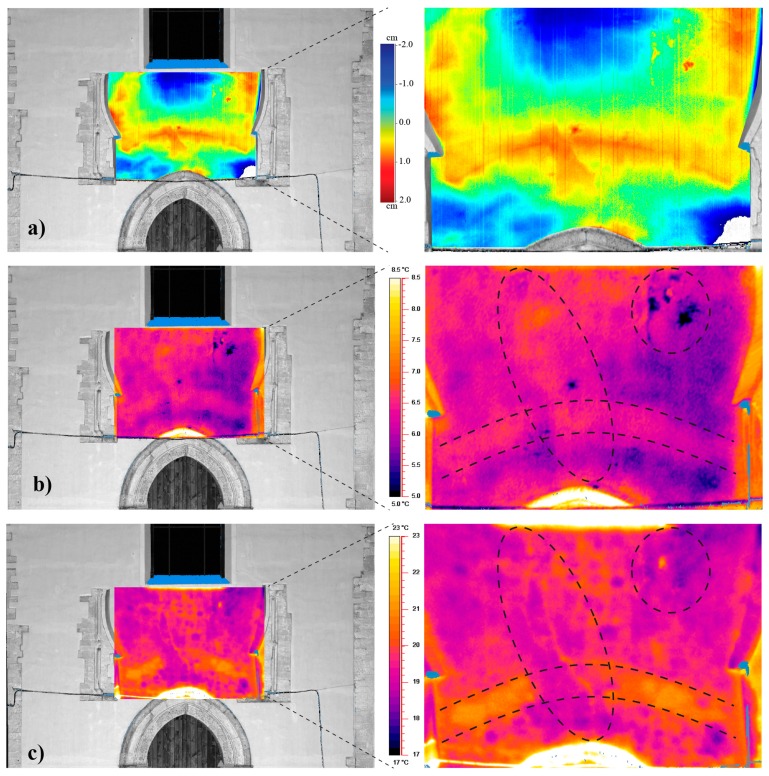
Particular of the façade of the church: (**a**) mesh by point cloud and map of the residuals with respect to a fitting plane; (**b**) image obtained in the evening of 4 December 2013 and (**c**) in the morning of 13 April 2014.

The map of the residuals of the façade, obtained as the distance between the point cloud acquired with a single scan and a best fitting plane, shows large irregularities in correspondence to the window (Figure 5c), where originally a rosette had been realized as reported by the historical survey. The same figure also shows deviations of the point on the façade localized immediately above the lancet arch, assuming the shape of a segmental arc linking the two columns (see area bounded by a dashed line in Figure 5c and in particular in Figure 6a).

A counterproof of the same anomaly can be detected by the thermal images, which, to permit the correlation between the two survey techniques, were projected over the mesh obtained by the point cloud (Figure 6b,c). In fact, the thermograms also allow one to observe an arch shape, similar to the one identified by laser scanning, showing a temperature of about 2 °C higher than the surrounding area. Different hypotheses can be made on the presence of the “hidden arch”: possibly another original element of the first building in the transition zone between the large portal and the now absent rosette, similar to those still exposed to the view, or a specific material used to protect the portal during the rebuilding work of the upper part of the façade.

To reduce systematic errors due to the irradiation variations and fluctuations, the testing procedures recommend the repetition of thermographic capture with different conditions of thermal exchange and a comparison of the temperatures of selected areas in the same framing at the same boundary conditions [11]. Therefore, in correspondence to the area between the top of the portal and the base of the window (Figure 6a), thermographic surveys have been executed during different days and times; the thermal images acquired on the evening of 4 December 2013 (Figure 6b) and the morning of 14 April 2014 (Figure 6c) are compared. The different lighting conditions make more obvious the hidden element and the cracks when sunlight is present; whereas, the detachment of plaster, although detectable in both of the moments, is better outlined in absence of the solar source. Although the anomalies are detectable in both thermograms, the differences between daylight thermal image and nighttime thermal image could be related with many factors, such as heat flux direction and different thermal resistance, due to the presence of different materials, and the air gaps [28]. Furthermore, in the comparison, the presence of lighthouses in the surrounding square, which produce a non-uniform source on the façade in the evening, influencing the recording by the thermal camera, must also be considered.

**Figure 7.**
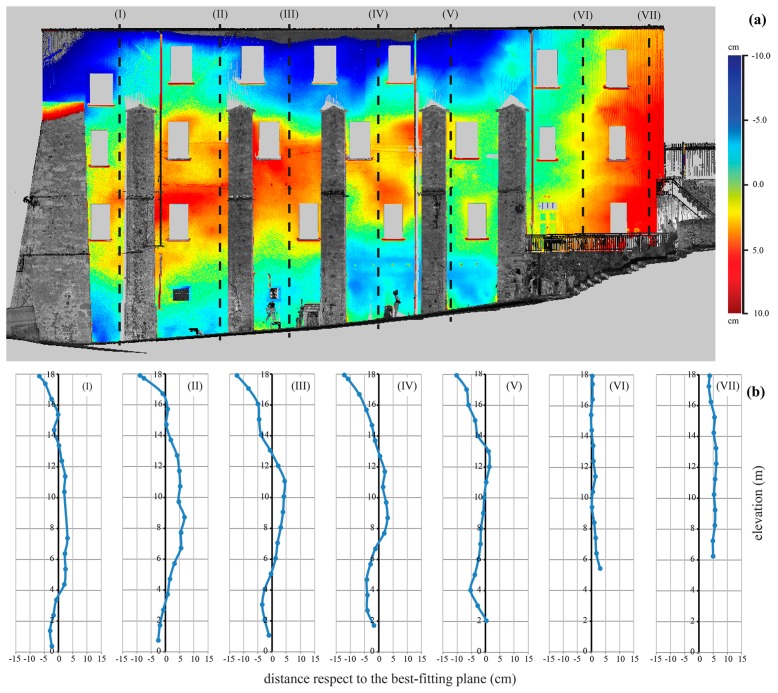
TLS survey of the external wall reinforced by buttresses: (**a**) point cloud and map of the residuals and (**b**) vertical profiles of the distance out of plain with respect to the reference plane.

Like the façade of the church, an analysis of the residuals with respect to a best-fitting plane was performed also for the external wall of the compound (Figure 7a), extended in direction SE-NW. The wall, with a length of about 35.1 m and a variable height between 17.7 m and 12.5 m, is supported by imposing buttresses extending from the base up to about 5 m from the top.

The deviation of points, with reference to a best fitting plane, permitted us to detect an extensive area of anomaly; in fact, in correspondence of the central part of wall, the figure shows the presence of significant positive out of plane deviations. These problems are probably due to ground settlement and they are related to the configuration of the structure and its transformations over time.

Analyzing the vertical profiles along some sections from SE to NW (Figure 7b), it is possible to note an accentuated deformed shape of the wall in the central part (see Sections I–V in the figure). Moreover, the NW part of wall, without buttresses, is almost shifted with respect to the fit plane (see Sections VI–VII in figure). The vertical profiles also show a relevant negative deviation in the upper part of the wall (more evident in Sections I–V); this behavior is conditioned by the presence of tension-rods put in place to reduce the tilting of the masonry.

**Figure 8.**
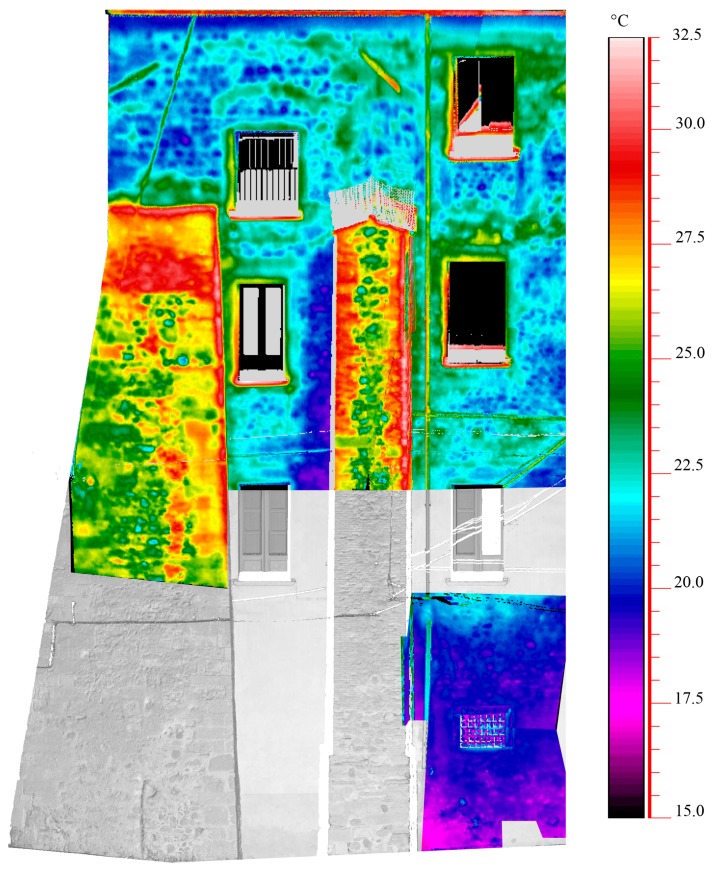
Thermograms projected on the point cloud relating to the external wall (represented up to Section II with reference to the Figure 7) of the St. Augustine Monumental Compound.

The thermographic survey obtained on the wall and the buttresses allowed us to detect high thermal differences. In Figure 8 some thermograms were projected on the point clouds of the wall. The temperature analysis demonstrates high local differences in the masonry. In fact, the wall was constructed by stones and covered by plastering, while the buttresses with made of composite material contaning both stones and bricks, which can be identified by the different detected temperatures, so probably these elements have been built in a subsequent period respect to the wall. This condition plays an important role in the evaluation of the mechanical strength to be assigned to the masonry. Moreover, a particular behavior can be found in correspondence with the openings, which have probably been changed over time. In the lower part of the wall the presence of moisture due to the capillary rise of water from the subsoil is detectable; indeed, the thermogram indicates the lowest temperatures.

**Figure 9.**
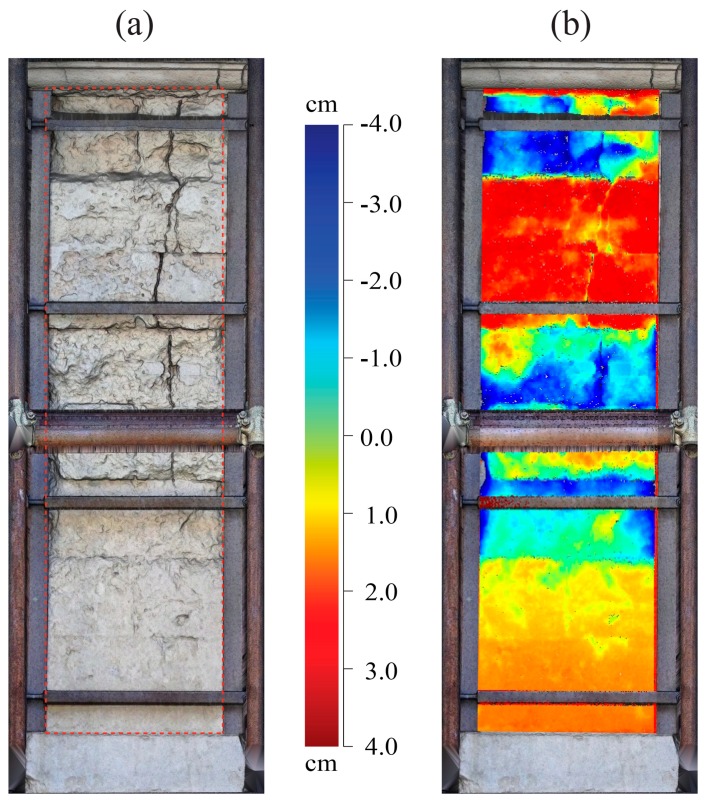
Results of the detailed analysis on a column of the inner colonnade: (**a**) mesh with extremely high resolution; (**b**) map of the residual respect with the best-fitting plane.

For the proposed case study, verifying the widespread instability of the inner colonnade, linked both to the misalignment of columns located at the different levels and to the cracks and degradation of the materials [29], the health state of this element was analyzed through the point clouds acquired by extremely high resolution of the TLS, scanned to distance less than 2 m with sub-millimeter resolution. The results, carried out for a surface of one of the most damaged columns, show the occurrence of cracks and a variable degradation of the stone material (Figure 9a). Additionally, is possible verify an in plane inclination of about 1.7 cm respect to the vertical axis, probably due to the eccentricity of loads. The map of the residuals, extracted for the same column with respect to the best-fitting plan, allowed us to outline with high accuracy the more degraded zones of the stone blocks (blue areas in Figure 9b); this phenomena induces a consequent reduction of the resistant section.

The detailed analysis on the overall instability of the colonnade and a comparison between the TLS survey and conventional measurement techniques was performed by the authors in previous works [29], to evaluate the consistency of the measurements. Summarizing, the most noteworthy finding is that the combined use of the two sensors, based on different operating principles, allows one to obtain better knowledge of the conservation state of historical buildings. In fact, with reference to the analyzed case history:

- the TLS survey permitted a complete 3D reconstruction of the building, in order to make a more accurate structural modeling and to provide the reference for the monitoring activities over time (cf. Figure 3);
- the combination of the data provided by both TLS and IR thermography allowed us to identify the anomalies on the masonry (cracks, detachments of plaster, moisture zones) and the different construction materials used during the initial construction and in the further phases of rebuilding and restoration (cf. Figures 4 and 5);
- the overlap of the thermal images on the point clouds and the comparison with the analysis of the residuals showed on the façade of the church, a large irregularity in correspondence to the window above the portal, where the historical survey suggests the existence of an ancient rosette (cf. Figure 5); in addition, on the wall reinforced by buttresses the deformed shape and the high heterogeneity of the construction materials was detected (cf. Figures 7 and 8);
- Moreover, the results on the church façade allowed us to also detect an anomaly immediately above the lancet arch of the portal, with a particular segmental arch shape, linked to a hidden element (cf. Figure 6).

## 5. Conclusions

The designed survey, combining both sensors, improved the knowledge of the conservation state of the monumental compound chosen as test building. Often the two sensors are used separately, decoupling the laser scanning data for 3D reconstruction and thermographic images to detect the anomalies; instead, the obtained results showed how the terrestrial laser scanner could be used as a diagnostic tool combined with other non-contact sensors, in order to study the architectural pathologies and to evaluate the vulnerability of historical buildings. In fact, following the proposed methodology, the systematic application of the data comparison allowed us to understand the potential of the integrated use of the two sensors, in order to recognize any decayed features. Moreover, it was possible to disclose hidden elements in the masonry and recognize zones characterized by different properties, probably caused by subsequent construction interventions in both ancient and recent times, therefore with different techniques and materials from the original ones.

The conceived methodology can be equally useful for the assessment of the health status of other historic buildings, allowing more detailed knowledge for purposes relating to conservation and restoration activities. In addition, the short time required for the data acquisition, the high potential of analysis and the significant results obtained for the presented case study suggest the adoption of this survey methodology in a protocol for the preservation of the architectural and cultural heritage against calamities. Furthermore, the application of this strategy could allow one to observe the progression of decay over time, confirming the possibility of monitoring the effects on ancient buildings due both to the permanent loads and accidental events, like earthquakes. Finally, the recorded data can characterize the screening phase to recognize those cases where further investigations, based on more time-consuming and expensive techniques, should be recommended.

A natural development of the research work is represented by the comparison of the results achieved by TLS, IR thermography and other non-destructive tests. In this context, the authors are already planning to study in depth the properties of the different materials of the historical compound, using not only the instruments described in this paper, but also further non-destructive tools, for example a field spectroradiometer in order to detect the signature of these materials over a wide spectral range.
